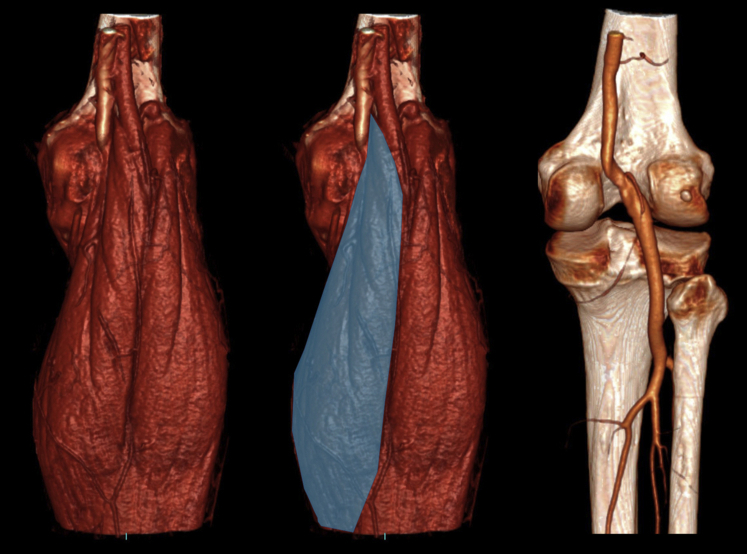# Cross-sectional imaging of popliteal artery entrapment syndrome

**DOI:** 10.1016/j.jvscit.2021.09.012

**Published:** 2021-10-13

**Authors:** Francesco Torella, Kimberly Lam, Simon Neequaye, Richard G. McWilliams

**Affiliations:** a Liverpool Vascular & Endovascular Service, Liverpool University Hospitals, Liverpool, United Kingdom; b Department of Radiology, Liverpool University Hospitals, Liverpool, United Kingdom

A 63-year-old asymptomatic man with unlimited exercise tolerance is investigated for suspected popliteal aneurysms, due to absent pedal pulses and prominent popliteal pulsations. Contrast-enhanced computed tomography (CT) shows occlusion of the left popliteal artery with geniculate collaterals surrounding the popliteal vein (*A*). The medial head of gastrocnemius separates the popliteal vein and the occluded (entrapped) popliteal artery. CT also shows entrapment of the right popliteal artery, due to extrinsic compression by the abnormally sited tendon of the medial head of gastrocnemius, which separates the artery and vein, mirroring the muscular anatomy on the left (*B*/Cover).

A second 55-year-old man, normally walking 10-12 miles per day without restriction, presents with acute right leg claudication at 200 m. Contrast-enhanced CT demonstrates left popliteal artery stenosis with post-stenotic dilatation and right distal popliteal embolic occlusion due to a popliteal aneurysm with thrombus secondary to bilateral popliteal entrapment syndrome (PAES) (*C*). Axial balanced flow fast field echo magnetic resonance images and corresponding CT axial images demonstrate that the left medial head of gastrocnemius passes between the popliteal artery and popliteal vein. However, the aneurysmal right popliteal artery and the right popliteal vein are both located medial to, and are not separated by, the medial head of gastrocnemius (*D*).

PAES is an uncommon cause of occlusive arterial disease, which is often bilateral. It is caused either by abnormal muscular insertions, typically of the medial head of gastrocnemius or the popliteus muscle, or by anomalous fibrous bands (types II, III, and IV, according to the modified Whelan classification).^1^ Less commonly, an abnormal course of the popliteal vessels around normally inserted muscles is responsible for the entrapment (type I). Arteriovenous (type V) and functional entrapments have also been described. As illustrated, cross-sectional studies are very useful in demonstrating the abnormal anatomy. These two cases also show that muscular abnormalities are not always symmetrical in bilateral PAES (all limbs presenting as type II, except for the right leg of the second patient, which can be classed as type I, due to the medial course of the artery around a normal insertion of the medial head of gastrocnemius).

Both patients consented to the publication of the case details and relative images.

**Figure:**
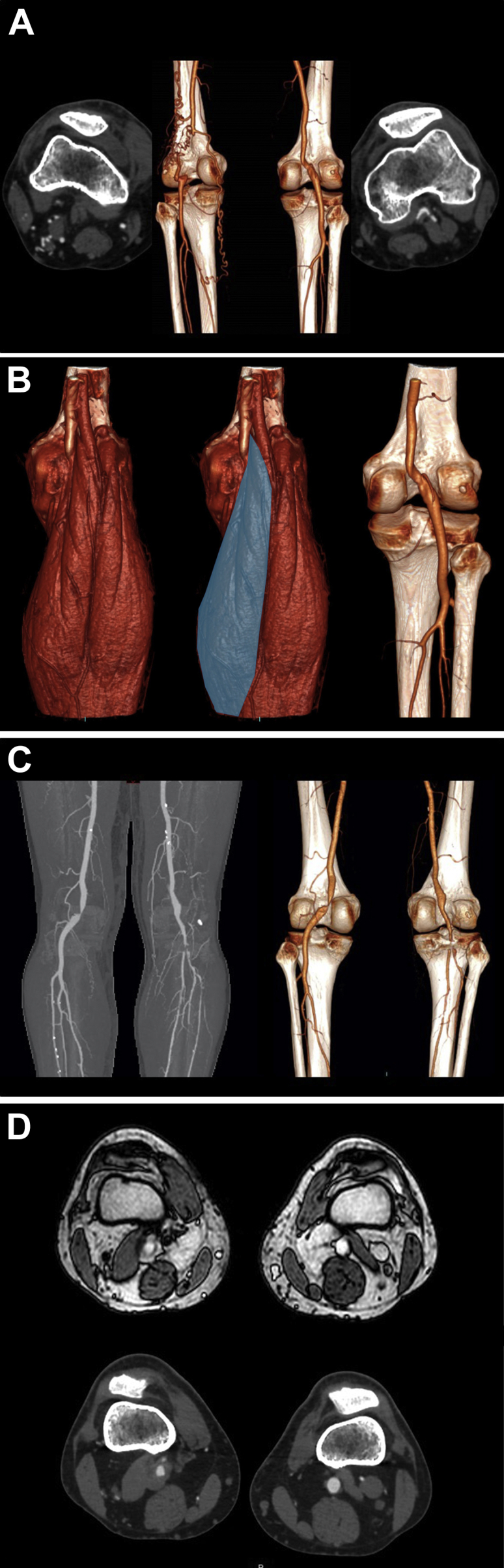

## Figures and Tables

**Cover image:**